# Artificial intelligence based prediction models for individuals at risk of multiple diabetic complications: A systematic review of the literature

**DOI:** 10.1111/jonm.13894

**Published:** 2022-11-23

**Authors:** Lucija Gosak, Kristina Martinović, Mateja Lorber, Gregor Stiglic

**Affiliations:** ^1^ Faculty of Health Sciences University of Maribor Maribor Slovenia; ^2^ Faculty of Health Sciences University of Primorska Izola Slovenia; ^3^ Faculty of Electrical Engineering and Computer Science University of Maribor Maribor Slovenia; ^4^ Usher Institute University of Edinburgh Edinburgh UK

**Keywords:** artificial intelligence, diabetes, prediction models, prediction of diabetes complications

## Abstract

**Aim:**

The aim of this review is to examine the effectiveness of artificial intelligence in predicting multimorbid diabetes‐related complications.

**Background:**

In diabetic patients, several complications are often present, which have a significant impact on the quality of life; therefore, it is crucial to predict the level of risk for diabetes and its complications.

**Evaluation:**

International databases PubMed, CINAHL, MEDLINE and Scopus were searched using the terms artificial intelligence, diabetes mellitus and prediction of complications to identify studies on the effectiveness of artificial intelligence for predicting multimorbid diabetes‐related complications. The results were organized by outcomes to allow more efficient comparison.

**Key issues:**

Based on the inclusion/exclusion criteria, 11 articles were included in the final analysis. The most frequently predicted complications were diabetic neuropathy (*n* = 7). Authors included from two to a maximum of 14 complications. The most commonly used prediction models were penalized regression, random forest and Naïve Bayes model neural network.

**Conclusion:**

The use of artificial intelligence can predict the risks of diabetes complications with greater precision based on available multidimensional datasets and provides an important tool for nurses working in preventive health care.

**Implications for Nursing Management:**

Using artificial intelligence contributes to a better quality of care, better autonomy of patients in diabetes management and reduction of complications, costs of medical care and mortality.

## 1 INTRODUCTION

Due to the increased morbidity in recent years, it is estimated that 642 million people will be diagnosed with diabetes in 2040 (Zou et al., 2018). Therefore, it is particularly crucial to assess and predict the level of risk for diabetes and its complications (Erandathi et al., 2020). Given the high prevalence, many researchers and physicians have developed various detection techniques based on artificial intelligence (AI) to approach problems better and avoid human error (Sharma & Shah, 2021). AI research in health care is rapidly accelerating in various fields of medicine (Kelly et al., 2019). The introduction of new technological advances will allow greater autonomy and more personalized treatment of patients (Briganti & Le Moine, 2020). AI has great potential to enhance the care of common chronic conditions significantly (Tarumi et al., 2021).

## 2 BACKGROUND

Managing chronic diseases is challenge for patients and health care providers (Tahri Sqalli & Al‐Thani, 2020). Diabetes mellitus (DM) is a chronic disease that is becoming increasingly worrying due to its high morbidity and is considered life‐threatening (Jian et al., 2021; Sharma & Shah, 2021). In DM2, it leads to an insulin resistance response, with a consequent decrease in insulin production. This occurs most often in people over 45 years of age (Goyal & Jialal, 2022). Eventually, it can affect any part of the body, causing serious complications (Jian et al., 2021), which can lead to multiple organ failures and have an impact on quality of life (Chaki et al., 2020; Ramesh et al., 2021). It is associated with a shorter life expectancy due to a higher risk of developing various diseases such as heart disease, stroke, blindness and amputation (Shah & Vella, 2014).

Diabetes‐related complications are a concern because they are unrecognized in the early stages of their development. Over time, they can become immutable and devastating, so identifying a high‐risk population and developing regular monitoring is crucial for prevention (Mosa et al., 2021; Ramesh et al., 2021). Complications range from acute complications, which are life‐threatening conditions (hypoglycemia or ketoacidosis), to chronic, longer‐lasting complications that can affect multiple organ systems (retinopathy, nephropathy, neuropathy, cardiovascular disease) (Nickerson & Dutta, 2012).

Patients with diabetes often have multimorbidity. The mentioned state describes several concomitant conditions with a significant impact on patient care and life quality (Chima et al., 2017). Predicting the development of disease complications is a demanding process due to the existence of unmeasured risk factors, unbalanced data, time‐varying dynamics data and various interventions for the disease (Yousefi & Tucker, 2020).

Accurate prediction of complications could help with more targeted measures to prevent or slow their development (Ljubic et al., 2020). AI with predictive analytics has great potential to improve the care of common chronic conditions with high morbidity and mortality and an important role in maintaining a healthy lifestyle, taking medication and monitoring glycemic status (Behera, 2021; Tarumi et al., 2021). AI is the use of computers and advanced technologies to simulate intelligent behaviour and critical thinking (Malik et al., 2019). It is often used to support health care staff to carry out tasks ranging from administrative work to patient monitoring (Bohr & Memarzadeh, 2020).

The use of AI can provide significant improvements in all areas of health care from diagnosis to treatment (Bohr & Memarzadeh, 2020). Studies show that AI methods are gradually being established as suitable for diabetes self‐management (Contreras & Vehi, 2018). The challenge for the use of AI in these areas of health care is not whether the technologies are effective and useful but to ensure that they are introduced into everyday clinical practice (Davenport & Kalakota, 2019). The use of AI would relieve the burden on health care professionals and increase the quality of work performed by reducing the possibility of errors and increasing accuracy (Aung et al., 2021).

The purpose of this systematic literature review is to determine the effectiveness of predicting multimorbid diabetes‐related complications with AI‐based models and determine which methods provide the best results in terms of prediction performance.

## 3 METHODS

The literature review was conducted according to the recommendations of Khan et al. (2003). In the first step, we addressed the review question: ‘Which AI‐based approaches are suitable for predicting multiple diabetes‐related complications?’. By answering this question, we have identified which techniques can help reduce the risk of complications and help diabetes care. In a second step, we set inclusion and exclusion criteria and restrictions for the selection of the literature, which are presented below. We used the keywords ‘artificial intelligence’, ‘diabetes mellitus type 2’, ‘prediction of complications’ and other synonyms (Table 1) in PubMed, CINAHL, MEDLINE and Scopus databases to search the literature. It needs to be noted that we only focus on AI‐based prediction models in this review, while other AI‐based approaches (e.g. AI‐based solutions in imaging or speech or text recognition or generation) are also present in the literature. The complete search strategy is shown in supporting information S1. The relevance and quality of the studies were assessed by two authors, who evaluated the eligibility of the articles based on predefined inclusion and exclusion criteria. In the last two steps, two authors extract the data from the articles and display them using an identification table. The results were then interpreted and discussed by all authors.

**TABLE 1:** Search strategy

#	Keywords
1	‘artificial intelligence’ OR AI OR ‘machine learning’ OR ‘deep learning’ OR ‘data mining’ OR ‘predictive models’ OR ‘predictive modelling’ OR ‘prediction model’ OR ‘neural network’ OR ‘deep learning’ OR ‘decision tree’ OR ‘random forest’ OR ‘nearest neighbours’ OR ‘support vector machines’ OR ‘gbm’ OR ‘gradient boosting’
2	‘diabetes mellitus type 2’ OR ‘type 2 diabetes’ OR ‘type 2 diabetes mellitus’ OR ‘noninsulin‐dependent diabetes’ OR ‘diabetes mellitus, noninsulin‐dependent’ OR ‘diabetes mellitus, noninsulin‐dependent’ OR ‘diabetes mellitus, noninsulin‐dependent’ OR ‘adult‐onset diabetes’ OR ‘diabetes mellitus, adult‐onset’ OR ‘diabetes mellitus, ketosis‐resistant’ OR ‘diabetes mellitus, maturity‐onset’ OR ‘diabetes mellitus, slow‐onset’ OR ‘diabetes mellitus, stable’ OR ‘maturity‐onset diabetes’ OR ‘maturity‐onset diabetes mellitus’ OR NIDDM OR T2DM OR ‘diabetic patient’
3	‘prediction of complications’ OR ‘prediction of diabetes complications’ OR ‘diabetes mellitus complications’ OR ‘retinopathy prediction’ OR ‘diabetic foot prediction’ OR ‘cardiovascular disease prediction’ OR ‘nephropathy prediction’ OR ‘neuropathy prediction’ OR ‘hypoglycemia prediction’ OR ‘hyperglycemia prediction’

To select relevant articles, we set the following inclusion criteria: (a) quantitative (e.g. case studies, randomized controlled trials, controlled trials), qualitative (e.g. interview, questionnaire, focus groups) studies and mixed‐method studies; (b) relating to the research topic of predicting multimorbid complications in diabetes using AI and (c) in the English language. Only studies with patients already diagnosed with diabetes were included in the study. Additionally, we included only studies where the prediction of multimorbid complications associated with diabetes was done using the AI techniques (support vector machines [SVMs], decision tree [DT], random forest [RF], gradient boosting [GBM], neural network [NN]). Articles using exclusively regression models were not included in this study, although some might argue that some elements of machine learning are also present in this type of typically used prediction models in health care. Usually, regression models were used as a baseline model for comparison with other AI‐based techniques. In such cases, we extracted the prediction performance results of the regression‐based prediction models as well. The results we aimed to extract from each study were areas under the curve (AUC) with corresponding confidence intervals (CIs) or standard deviations (SD). Since some studies did not report the AUC results, we also extracted accuracy, sensitivity and specificity where available. The exclusion criteria were as follows: (a) other types of surveys, such as cross‐sectional, observational surveys, summaries, commentaries, protocols and cohort studies; (b) duplicates between databases; and (c) articles that do not include AI‐based prediction models and do not predict multimorbid complications as a consequence of diabetes. We did not include any publication date limits when searching for the articles.

Two authors assessed the adequacy of the studies based on inclusion and exclusion criteria. In case of disagreement, we resolved this by discussion between the authors. The two authors also extracted key results and information from the articles and presented them in tabular form (Table 2 and supporting information S2).

**TABLE 2:** Characteristics of the included studies

Authors (year)	Types of AI	Sample size	Data source	Diabetes‐related complications	Findings
Aminian et al. (2020)	Regression (Cox proportional hazards, exponential and fine‐grey) and random forest (RF)	287.438	Electronic health record from the Cleveland Clinic between 1998 and 2017.	All‐cause mortality, coronary artery events, heart failure, nephropathy	The most important variables that contributed to the mortality model were age, BMI, history of heart failure, insulin use and smoking status. The IDC risk scores were better than RECODe in all outcomes examined, in terms of IPA, AUC and calibration. The final model is used as the IDC risk calculator, the online version of which is freely available at https://riskcalc.org or as a smartphone mobile app called BariatricCalc.
Dagliati et al. (2018)	Logistic regression (LR), naïve Bayes (NB), support vector machines (SVMs) and random forest (RF)	943	From electronic medical records collected by IRCCS (Istituto di Ricovero e Cura a Carattere Scientifico, meaning research hospital), Istituto Clinico Scientifico Maugeri (ICSM), Pavia Hospital, Italy.	Nephropathy, neuropathy, retinopathy	Authors recommend the use of the LR models as the difference in performance was not significant, while on the other hand, LR models provide a clear implementation of the coefficient values and can be presented in the graphical form as nomograms. The variables considered in the model were sex, age, time since diagnosis, body mass index, glycated haemoglobin, hypertension and smoking. Although AUC values were higher for SVM and RF when the data sets were balanced, SVM and RF models are more difficult to interpret in clinical practice. The final models, adjusted for complications, provided accuracies up to 0.838.
Fan et al. (2021)	Artificial neural network (ANN), Bayesian network (BN), chi‐squared automatic interaction detector (CHAID), classification and regression tree (CRT), quick unbiased efficient statistical tree (QUEST), discriminate (D) and ensemble (XF) models	129	The data used were patient data collected at Sichuan Provincial People's Hospital from January 2010 to December 2015.	Diabetic nephropathy (DN), diabetic peripheral neuropathy (DPN), diabetic angiopathy (DA), diabetic eye disease (DED)	The variables that had the biggest impact were age, duration of type 2 diabetes, types of insulin and duration of unadjusted hypoglycaemic treatment. Of the 18 models used in the study, most were effective. The ensemble model was the best among the models for predicting diabetic nephropathy and diabetic angiopathy, and the discriminate model was the best among the models for predicting diabetic peripheral neuropathy and diabetic eye disease. The authors state that the developed models, after validation and screening, can be used for clinical practice for patients with type 2 diabetes and for health care professionals.
Kim et al. (2019)	Least absolute shrinkage and selection operator (LASSO), gradient boosting machine (GBM)	81.091	Sourced from OptumLabs® Data Warehouse.	Ischemic heart disease (IHD), congestive heart failure (CHF), cerebrovascular disease (CVD), peripheral vascular disease (PVD), nephropathy	The model used in the research is learned from the national cohort and is externally validated, thus contributing to the transferability of the model. Four different models have been built. In internal validation, the predictive performance was very similar between the models. The model was implemented in two local health systems, the University of Minnesota Medical Center and the Mayo Clinic for external evaluation.
Lagani et al. (2015)	Cox regression, ridge Cox regression, accelerated failure time (AFT), random survival forest (RSF) and support vector machine censored regression (SVCR)	1.441	The data come from research diabetes control and complications trial (DCCT) in epidemiology of diabetes interventions and complications (EDIC).	Retinopathy, hypoglycemia, neuropathy, ketoacidosis, microalbuminuria, proteinuria	The model results range from 0.6024 to 0.8333, which means that they all have comparable predictive power. Future work by the authors will focus on validating the models to further strengthen the results obtained.
Lee et al. (2021)	Random survival forests (RSF), univariate Cox regression	25.186	Data on patients with type 1 and type 2 diabetes prescribed insulin in Hong Kong public hospital outpatient clinics from 1 January to 31 December 2009.	Mortality, renal, peripheral vascular disease (PVD), neurological, ophthalmological, ischemic stroke, atrial fibrillation (AF), heart failure (HF), intracranial haemorrhage (ICH), ischemic heart disease (IHD), acute myocardial infarction (AMI), osteoporosis, dementia	The authors of the article note that machine learning algorithms can further improve the prediction of the risks of an event occurring in patients with diabetes. According to the C‐index evaluation, the model outperforms both RSF and Cox for mortality survival analysis. The model also shows higher prediction accuracy.
Liu et al. (2020)	Bayesian network model (BN), bootstrap and Tabu search algorithm, Markov blanket (MB), decision tree model, Naïve Bayes model (NB), random Forest model (RF) and C5.0	1.485	The data were collected from the National Clinical Centre for Health between 1 January 2009 and 31 December 2009.	Diabetic nephropathy (DN), diabetic foot (DF), diabetic macrovascular complications (DMV), diabetic peripheral neuropathy (DPN), diabetic ketoacidosis (DK)	The BN model was selected as the best model based on the results and can be used as a general tool for disease prevention, monitoring and management. The BN model is effective for predicting diabetic nephropathy, diabetic foot, diabetic macrovascular complications and diabetic ketoacidosis.
Ljubic et al. (2020)	Advanced machine learning algorithms, recurrent neural network (RNN), long short‐term memory (LSTM) and RNN gated recurrent unit (GRU RRN), bidirectional GRU, deep learning methods, random forest (RF) and multilayer perceptron (MLP)	*Four visits*: 19.589 *Three visits*: 26.973 *Two visits*: 26.973	Data are derived from state‐level inpatient databases pertaining to health care costs and utilization in California for the period 2003 to 2011.	Angina pectoris	Based on the results, the authors concluded that it is the best of the analysed RNN GRU models, followed by the RNN LSTM. The RNN GRU model was more accurate (73%–83% vs. 66%–76%) compared with traditional models. RNN models were most accurate in predicting depressive disorder and IHD. The authors suggest that the models could be integrated into a clinical decision support system.
		*Four visits*: 26.973 *Three visits*: 44.688 *Two visits*: 62.016		Atherosclerosis	
		*Four visits*: 52.959 *Three visits*: 81.658 *Two visits*: 147.718		Ischemic chronic heart disease (ICHD)	
		*Four visits*: 56.343 *Three visits*: 78.732 *Two visits*: 135.492		Depressive disorder	
		*Four visits*: 8.576 *Three visits*: 12.030 *Two visits*: 16.884		Hearing impairment	
		*Four visits*: 38.380 *Three visits*: 52.896 *Two visits*: 92.961		Myocardial infarction (MI)	
		*Four visits*: 37.982 *Three visits*: 52.283 *Two visits*: 71.053		Nephropathy	
		*Four visits*: 49.060 *Three visits*: 69.053 *Two visits*: 99.825		Neuropathy	
		*Four visits*: 48.565 *Three visits*: 67.686 *Two visits*: 93.905		Peripheral vascular disease (PVD)	
		*Four visits*: 27.796 *Three visits*: 36.221 *Two visits*: 58.641		Retinopathy	
Ozdemir et al. (2020)	Support vector machines (SVMs), extreme learning machines (ELMs) and artificial neural networks (ANNs)	72	It is not specified where the data are taken from. The data were taken from patients aged 30 years or older with a diagnosis of type 2 diabetes.	Neuropathy, neuropathic pain, kinesiophobia	Using computer‐assisted clinical decision support systems, it can be effective in managing complications and motor dysfunctions.
Shi et al. (2020)	LASSO regression method, logistic regression	4.219	Data obtained from a questionnaire, physical examination and biochemical tests of diabetic patients in Shanghai.	Diabetic nephropathy (DN), diabetic retinopathy (DR)	The authors found a moderately good differentiation and ability to confirm and predict the incidence of diabetic nephropathy and diabetic retinopathy. Seven variables were included in the logistic regression: disease course, BMI, TG, SBP, PBG, HbA1c and BUN.
Wang et al. (2021)	Multilabel algorithms (BR, RankSVM, ML‐KNN, ML‐RBF, BP‐MLL and WML‐SSLM)	17.300	Not available.	Macrovascular, microvascular, neuropathy	The authors propose a WML‐SSLM model to predict complications due to diabetes.

Abbreviation: AI, artificial intelligence.

## 4 RESULTS

**FIGURE 1:**
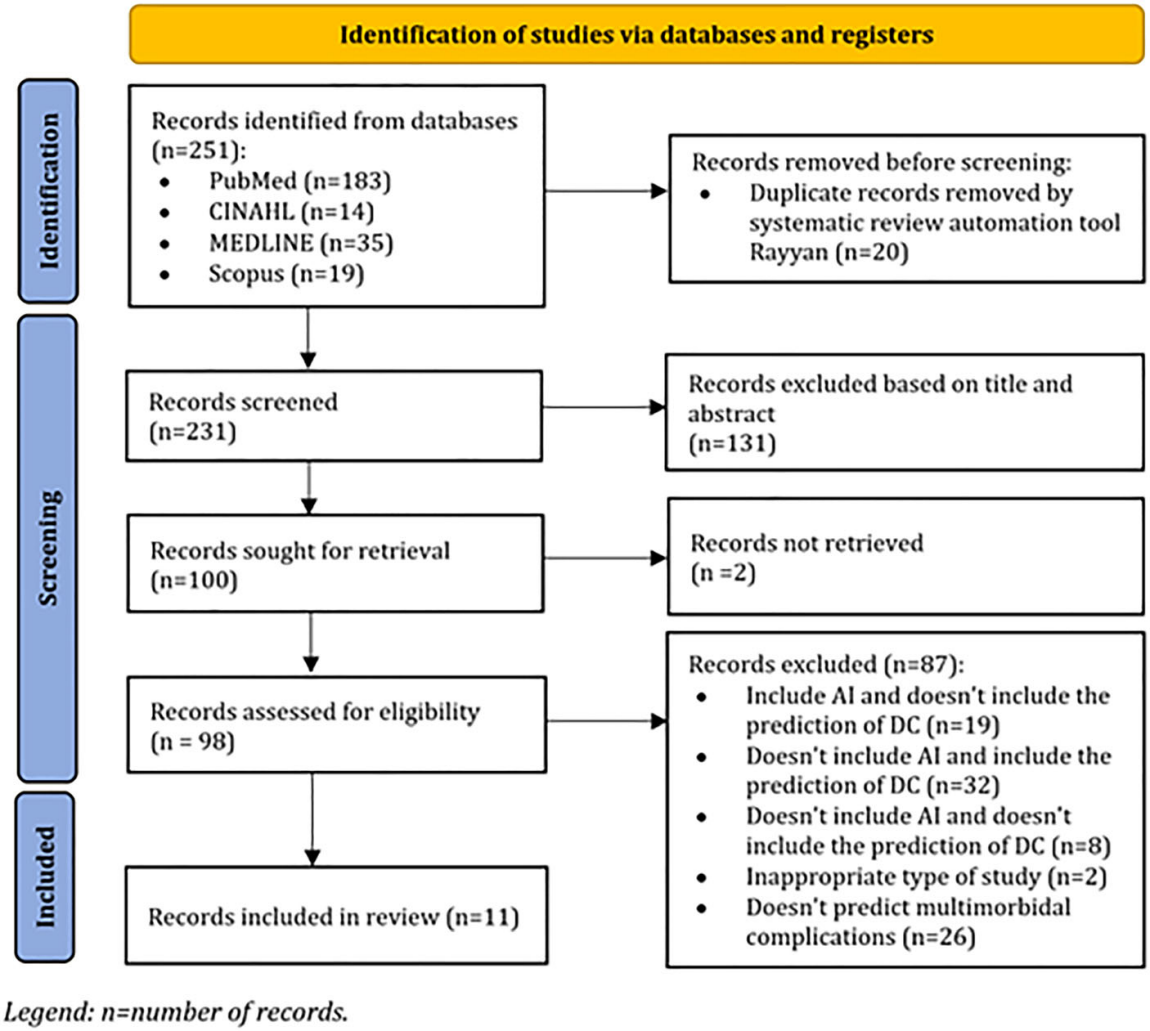
Flow diagram for systematic reviews

Based on the search string, we found a total of 251 results in the databases. Of these, 38 articles included the prediction of diabetes‐related complications, but only 11 hits included studies where two or more complications were predicted (Figure 1).

Table 2 presents the characteristics of the included studies.

**FIGURE 2:**
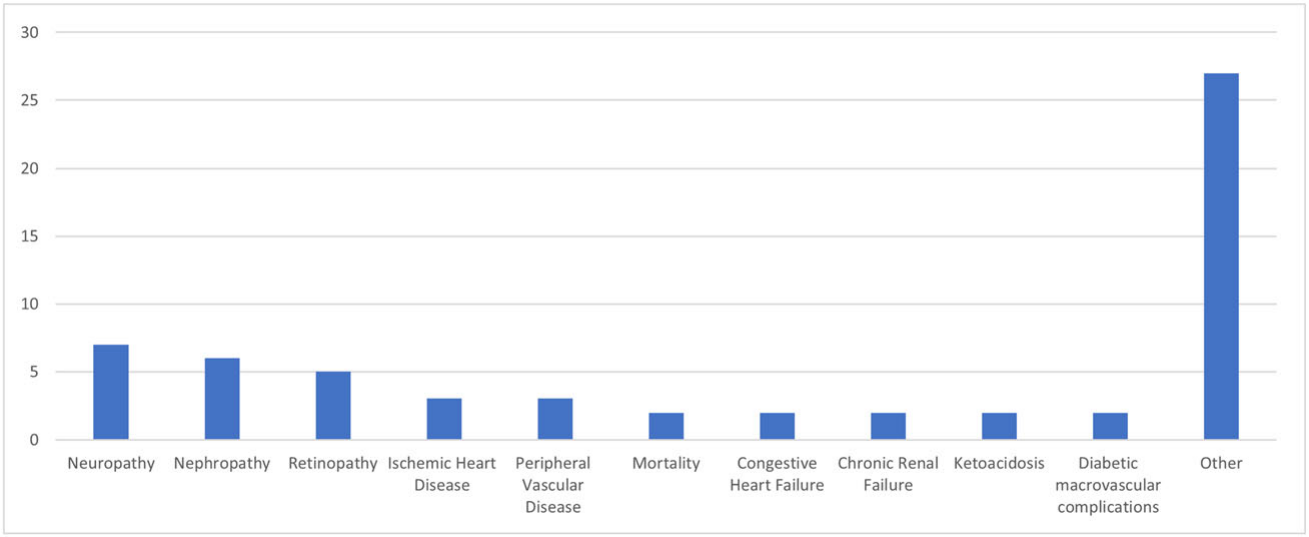
The most commonly treated complications of diabetes

Supporting information S2 presents the prediction performance results of the identified models (AUC, CI, accuracy, sensitivity, specificity) for predicting diabetes‐related complications. Based on our systematic review, we found that models most often predict the risk of developing diabetic neuropathy (*n* = 7) (Dagliati et al., 2018; Fan et al., 2021; Lagani et al., 2015; Liu et al., 2020; Ljubic et al., 2020; Ozdemir et al., 2020; Wang et al., 2021) and diabetic nephropathy (*n* = 6). Slightly less frequently, the authors predicted the development of diabetic retinopathy (*n* = 5) (Figure 2). The most commonly predicted complication in the models, diabetic neuropathy, is also one of the most common complications of diabetes, leading to loss of sensory function in the patient (Feldman et al., 2019; Juster‐Switlyk & Smith, 2016).

Patients diagnosed with type 2 diabetes are often at risk of developing multiple additional comorbidities (Cicek et al., 2021). Therefore, in our systematic review, we focused on models that allow the user to predict multiple complications. The model included at least two complications and up to 14 different complications or conditions caused by diabetes type 2.

The most common types of AI used by the authors in their work are regression, RF, Naïve Bayes and NN‐based models. Dagliati et al. (2018) reported AUC values for three different models for three different forecast periods of 3, 5 and 7 years. We compared the AUCs for the 3‐year forecasting models, and the AUCs were 0.489 for the SVM model, 0.5 for the RF model, 0.726 for the LR and 0.533 for the NB model. Accuracy was reported only for LR. SD or CIs were not reported. Fan et al. (2021) reported AUC of 0.847 ± 0.081 for XF, 0.787 ± 0.081 for CHAID and 0.720 ± 0.060 for QUEST. Lagani et al. (2015) reported AUC for predicting neuropathy in both type 1 and type 2 diabetes. T1DM (internal) was 0.6661, T1DM (external) was 0.735 and T2DM (external) was 0.4359. Liu et al. (2020) presented the AUC for the following models: BN (0.545), BN‐wopi (0.685), NB (0.505), RF (0.516) and C5.0 (0.527). Ljubic et al. (2020) did not present the AUC in their paper but only gave the accuracy for all models at four, three and two visits. We present a comparison for four visits only and the values for the GRU RNN model with the accuracy of 0.746 ± 0.053, bidirectional GRU with 0.746 ± 0.053, 1‐way LSTM with 0.719 ± 0.073, RF with 0.671 ± 0.033 and MLP with 0.668 ± 0.039. Ozdemir et al. (2020) reported the same result for AUC and accuracy resulting in an almost perfect classification performance of 0.99. Since no data or code was available, it is difficult to argue the robustness of their evaluation protocol. Wang et al. (2021) reported only accuracy. Multilabel classifier metric was used to estimate the accuracy of covering all three complications at once. BR (linear) resulted in accuracy of 0.685 ± 0.000, BR (poly) of 0.701 ± 0.000, BR (rbf) of 0.701 ± 0.000, WML‐SSLM (linear) of 0.520 ± 0.001, WML‐SSLM (poly) of 0.661 ± 0.002 and WML‐SSLM (rbf) of 0.697 ± 0.002. If we exclude extremely optimistic results from Ozdemir et al. (2020), we can conclude with the observation that prediction performance in terms of AUC for the best model in the study ranges from 0.685 up to 0.847. However, one needs to be aware that different studies employ different approaches to cross‐validation and internal versus external evaluation which can influence the reliability of the reported results. In many cases, the description of the evaluation protocol is weak or even nonexistent.

Aminian et al. (2020) considered 26 different baseline variables (demographics, medical history, laboratory data, medications) for prediction in their study. In addition to classical regression models, machine learning approaches were also used. They found that cardiovascular events are among the most frequent complications in patients with type 2 diabetes and obesity. Dagliati et al. (2018) considered the following variables: sex, age, time since diagnosis, body mass index, glycated haemoglobin, hypertension and smoking status. LR after RF imputation identified risk factors for microvascular complications. Fan et al. (2021) used age, sex, duration of diabetes and duration of unadjusted treatment, insulin, the total cost of medications and number of medications, genetic history of diabetes and dyslipidemia. XF was the best performing model for predicting diabetic nephropathy and diabetic angiopathy, D for predicting diabetic neuropathy and diabetic eye disease and BN for HbA1c (Fan et al., 2021). Fifty‐one clinical variables were used in the selected predictive models and included between five and fifteen risk factors depending on the specific outcome (Lagani et al., 2015). Lee et al. (2021) found that higher HbA1c and lipid measurements were associated with an increased risk of complications and various comorbidities. Ljubic et al. (2020) cite the RNN GRU model as the most accurate model, followed by the RNN LSTM model.

## 5 DISCUSSION

When patients have additional chronic comorbid conditions, there is an almost exponential increase in the cost of care related to health care services, medicines and hospital admissions (McPhail, 2016). Complications resulting from type 2 diabetes, such as nephropathy, neuropathy, blindness, cardiovascular disease and amputations reduce their quality of life and increase mortality. With advances in the care and treatment of type 2 diabetes and its complications, people with diabetes can live with their condition for longer (Deshpande et al., 2008; Liu et al., 2010). It is important to detect the development of complications early enough, as rapid action can prevent or delay the onset of chronic complications (Marshall & Flyvbjerg, 2006). AI plays an important role in predicting complications using basic clinical and biochemical patient data, but predicting the occurrence of different complications is a challenging task due to different risk factors, unbalanced data and rapid changes (Singla et al., 2019). Therefore, there is an increasing emphasis on the use of appropriate AI techniques to predict prognosis (Singla et al., 2019; Yousefi & Tucker, 2020). Consequently, accurate prediction helps to target nursing interventions better (Ljubic et al., 2020). AI supports nurses in clinical decision‐making and other tasks that are not directly related to the patient (Seibert et al., 2021).

Nurses, as the largest part of all workers involved in health systems, will benefit enormously from AI (Shang, 2021). The role of nurses is to be actively involved in decision‐making regarding the implementation of AI in the health care system and to ensure that they ensure that these changes are implemented in accordance with the ethical principles and values of nursing (Buchanan et al., 2020). With the introduction of technology, nurses' experience, knowledge and skills will be transformed into learning new ways of thinking and processing information (Robert, 2019). Our literature review also found that nurses are rarely involved in the interdisciplinary team that carries out the implementation. In most cases, it was individual research carried out by the researchers. It would be important to involve health care providers as they can influence the actual implementation of AI in clinical practice.

IT skills training should be offered to nursing students and those already working in a clinical setting (Risling, 2017). It is important that they understand the potential of AI and its impact on health care (Fritz & Dermody, 2019). It is also important that nurses are empowered by technological change and that they are not just passively involved in it (Ng et al., 2021).

In practice, there is still a lack of models or frameworks for implementing AI in everyday health care practices (Svedberg et al., 2022). Yet, there are individual gaps in the literature on AI in nursing, with implications for clinical practice (Shang, 2021). The content of research in the field is very diverse, so it is important to develop guidelines on research reporting and technology implementation (von Gerich et al., 2021). This is also the problem we encountered in our literature review, and it is also the biggest limitation. The included research reported different methodological approaches and, above all, reported different results that cannot be synthesized due to inequalities. Despite the heterogeneity of the studies, most of them were based on data from patients diagnosed with diabetes, extracted from various electronic records. It is also very difficult to compare individual studies with each other, as they included a wide range of population sizes (minimum 129, maximum 287,438).

## 6 CONCLUSIONS

New strategies are necessary to empower people worldwide to prevent and manage diabetes. Diabetes management requires constant monitoring and recommendations. AI provides multiple approaches for preventing and managing various chronic diseases, also as diabetes. The use of AI can predict risks of diabetes complications with greater precision based on available multidimensional datasets and provides a model for AI‐assisted prognosis and diagnosis of next‐generation diabetes complications.

## 7 IMPLICATIONS FOR NURSING MANAGEMENT

By using AI methods, we can help facilitate the control of diabetes and detect the presence of risk in patients for the development of multimorbid complications promptly. In this way, we contribute to a better quality of care, better autonomy of patients in the course of treatment of their disease and reduction of complications, costs of medical care and mortality. The use of AI methods also serves as a tool for nurses when working with patients, making it easier for them to predict disease progression and thus contributing better preventive care for patients.

## CONFLICT OF INTEREST

The authors declare no conflict of interest.

## ETHICS STATEMENT

Ethical approval was not required as the research does not involve any participants and only involves a review of the literature.

## AUTHOR CONTRIBUTIONS

*Conceptualization:* Lucija Gosak and Gregor Stiglic. *Data analysis:* Lucija Gosak, Gregor Stiglic, Mateja Lorber and Kristina Martinović. *Methodology:* Lucija Gosak, Gregor Stiglic, Mateja Lorber and Kristina Martinović. *Supervision:* Lucija Gosak and Gregor Stiglic. *Writing (original draft):* Lucija Gosak and Gregor Stiglic. *Writing (review):* Lucija Gosak, Gregor Stiglic, Mateja Lorber and Kristina Martinović. All authors have read and agreed to the published version of the manuscript.

## Supporting information


**Table S1.** Search strategy (01.11.2021)
**Table S2.** Outcomes of models for predicting diabetes‐related complications

## Data Availability

The data that support the findings of this study are available in the supporting information of this article.

## References

[jonm13894-bib-0001] Aminian, A. , Zajichek, A. , Arterburn, D. E. , Wolski, K. E. , Brethauer, S. A. , Schauer, P. R. , Nissen, S. E. , & Kattan, M. W. (2020). Predicting the 10‐year risk of end‐organ complications of type 2 diabetes with and without metabolic surgery: A machine learning approach. Diabetes Care, 43(4), 852–859. 10.2337/dc19-2057 32029638 PMC7646205

[jonm13894-bib-0002] Aung, Y. Y. , Wong, D. C. , & Ting, D. S. (2021). The promise of artificial intelligence: A review of the opportunities and challenges of artificial intelligence in healthcare. British Medical Bulletin, 139(1), 4–15. 10.1093/bmb/ldab016 34405854

[jonm13894-bib-0003] Behera, A. (2021). Use of artificial intelligence for management and identification of complications in diabetes. Clinical Diabetology, 10(2), 221–225. 10.5603/DK.a2021.0007

[jonm13894-bib-0004] Bohr, A. , & Memarzadeh, K. (2020). The rise of artificial intelligence in healthcare applications. In Artificial intelligence in healthcare (pp. 25–60). Academic Press. 10.1016/B978-0-12-818438-7.00002-2

[jonm13894-bib-0005] Briganti, G. , & Le Moine, O. (2020). Artificial intelligence in medicine: Today and tomorrow. Frontiers in Medicine, 7, 27. 10.3389/fmed.2020.00027 32118012 PMC7012990

[jonm13894-bib-0006] Buchanan, C. , Howitt, M. L. , Wilson, R. , Booth, R. G. , Risling, T. , & Bamford, M. (2020). Predicted influences of artificial intelligence on the domains of nursing: Scoping review. JMIR Nursing, 3(1), e23939. 10.2196/23939 34406963 PMC8373374

[jonm13894-bib-0007] Chaki, J. , Ganesh, S. T. , Cidham, S. K. , & Theertan, S. A. (2020). Machine learning and artificial intelligence‐based diabetes mellitus detection and self‐management: A systematic review. Journal of King Saud University‐Computer and Information Sciences., 34, 3204–3225. 10.1016/j.jksuci.2020.06.013

[jonm13894-bib-0008] Chima, C. C. , Salemi, J. L. , Wang, M. , de Grubb, M. C. M. , Gonzalez, S. J. , & Zoorob, R. J. (2017). Multimorbidity is associated with increased rates of depression in patients hospitalized with diabetes mellitus in the United States. Journal of Diabetes and its Complications, 31(11), 1571–1579. 10.1016/j.jdiacomp.2017.08.001 28893494

[jonm13894-bib-0009] Cicek, M. , Buckley, J. , Pearson‐Stuttard, J. , & Gregg, E. W. (2021). Characterizing multimorbidity from type 2 diabetes: Insights from clustering approaches. Endocrinology and Metabolism Clinics, 50(3), 531–558. 10.1016/j.ecl.2021.05.012 34399960 PMC8383848

[jonm13894-bib-0010] Contreras, I. , & Vehi, J. (2018). Artificial intelligence for diabetes management and decision support: A literature review. Journal of Medical Internet Research, 20(5), e10775. 10.2196/10775 29848472 PMC6000484

[jonm13894-bib-0011] Dagliati, A. , Marini, S. , Sacchi, L. , Cogni, G. , Teliti, M. , Tibollo, V. , de Cata, P. , Chiovato, L. , & Bellazzi, R. (2018). Machine learning methods to predict diabetes complications. Journal of Diabetes Science and Technology, 12(2), 295–302. 10.1177/1932296817706375 28494618 PMC5851210

[jonm13894-bib-0012] Davenport, T. , & Kalakota, R. (2019). The potential for artificial intelligence in healthcare. Future Healthcare Journal, 6(2), 94–98. 10.7861/futurehosp.6-2-94 PMC6616181 31363513

[jonm13894-bib-0013] Deshpande, A. D. , Harris‐Hayes, M. , & Schootman, M. (2008). Epidemiology of diabetes and diabetes‐related complications. Physical Therapy, 88(11), 1254–1264. 10.2522/ptj.20080020 18801858 PMC3870323

[jonm13894-bib-0014] Erandathi, M. A. , Wang, W. Y. , & Mayo, M. (2020, August). Predicting diabetes mellitus and its complications through a graph‐based risk scoring system. Proceedings of the 4th International Conference on Medical and Health Informatics, 1–7.

[jonm13894-bib-0015] Fan, Y. , Long, E. , Cai, L. , Cao, Q. , Wu, X. , & Tong, R. (2021). Machine learning approaches to predict risks of diabetic complications and poor glycemic control in nonadherent type 2 diabetes. Frontiers in Pharmacology, 12, 665951. 10.3389/fphar.2021.665951 34239440 PMC8258097

[jonm13894-bib-0016] Feldman, E. L. , Callaghan, B. C. , Pop‐Busui, R. , Zochodne, D. W. , Wright, D. E. , Bennett, D. L. , & Viswanathan, V. (2019). Diabetic neuropathy. Nature Reviews. Disease Primers, 5(1), 1–18. 10.1038/s41572-019-0097-9 PMC7096070 31197183

[jonm13894-bib-0017] Fritz, R. L. , & Dermody, G. (2019). A nurse‐driven method for developing artificial intelligence in “smart” homes for aging‐in‐place. Nursing Outlook, 67(2), 140–153. 10.1016/j.outlook.2018.11.004 30551883 PMC6450732

[jonm13894-bib-0018] Goyal, R. , & Jialal, I. (2022). Diabetes mellitus type 2. StatPearls [Internet]. https://www.ncbi.nlm.nih.gov/books/NBK513253/

[jonm13894-bib-0019] Jian, Y. , Pasquier, M. , Sagahyroon, A. , & Aloul, F. (2021). A machine learning approach to predicting diabetes complications. Healthcare, 9(12), 1712. 10.3390/healthcare9121712 34946438 PMC8702133

[jonm13894-bib-0020] Juster‐Switlyk, K. , & Smith, A. G. (2016). Updates in diabetic peripheral neuropathy. F1000Research, 5, 738. 10.12688/f1000research.7898.1 PMC4847561 27158461

[jonm13894-bib-0021] Kelly, C. J. , Karthikesalingam, A. , Suleyman, M. , Corrado, G. , & King, D. (2019). Key challenges for delivering clinical impact with artificial intelligence. BMC Medicine, 17(1), 1, 195–9. 10.1186/s12916-019-1426-2 31665002 PMC6821018

[jonm13894-bib-0022] Khan, K. S. , Kunz, R. , Kleijnen, J. , & Antes, G. (2003). Five steps to conducting a systematic review. Journal of the Royal Society of Medicine, 96(3), 118–121. 10.1258/jrsm.96.3.118 12612111 PMC539417

[jonm13894-bib-0023] Kim, E. , Caraballo, P. J. , Castro, M. R. , Pieczkiewicz, D. S. , & Simon, G. J. (2019). Towards more accessible precision medicine: Building a more transferable machine learning model to support prognostic decisions for micro‐and macrovascular complications of type 2 diabetes mellitus. Journal of Medical Systems, 43(7), 1–12. 10.1007/s10916-019-1321-6 31098679

[jonm13894-bib-0024] Lagani, V. , Chiarugi, F. , Thomson, S. , Fursse, J. , Lakasing, E. , Jones, R. W. , & Tsamardinos, I. (2015). Development and validation of risk assessment models for diabetes‐related complications based on the DCCT/EDIC data. Journal of Diabetes and its Complications, 29(4), 479–487. 10.1016/j.jdiacomp.2015.03.001 25772254

[jonm13894-bib-0025] Lee, S. , Zhou, J. , Wong, W. T. , Liu, T. , Wu, W. K. , Wong, I. C. K. , Zhang, Q. , & Tse, G. (2021). Glycemic and lipid variability for predicting complications and mortality in diabetes mellitus using machine learning. BMC Endocrine Disorders, 21(1), 1–15. 10.1186/s12902-021-00751-4 33947391 PMC8097996

[jonm13894-bib-0026] Liu, S. , Zhang, R. , Shang, X. , & Li, W. (2020). Analysis for warning factors of type 2 diabetes mellitus complications with Markov blanket based on a Bayesian network model. Computer Methods and Programs in Biomedicine, 188, 105302. 10.1016/j.cmpb.2019.105302 31923820

[jonm13894-bib-0027] Liu, Z. , Fu, C. , Wang, W. , & Xu, B. (2010). Prevalence of chronic complications of type 2 diabetes mellitus in outpatients‐a cross‐sectional hospital based survey in urban China. Health and Quality of Life Outcomes, 8(1), 1–9. 10.1186/1477-7525-8-62 20579389 PMC2906445

[jonm13894-bib-0028] Ljubic, B. , Hai, A. A. , Stanojevic, M. , Diaz, W. , Polimac, D. , Pavlovski, M. , & Obradovic, Z. (2020). Predicting complications of diabetes mellitus using advanced machine learning algorithms. Journal of the American Medical Informatics Association, 27(9), 1343–1351. 10.1093/jamia/ocaa120 32869093 PMC7647294

[jonm13894-bib-0029] Malik, P. , Pathania, M. , & Rathaur, V. K. (2019). Overview of artificial intelligence in medicine. Journal of Family Medicine and Primary Care, 8(7), 2328–2331. 10.4103/jfmpc.jfmpc_440_19 PMC6691444 31463251

[jonm13894-bib-0030] Marshall, S. M. , & Flyvbjerg, A. (2006). Prevention and early detection of vascular complications of diabetes. BMJ, 333(7566), 475–480. 10.1136/bmj.38922.650521.80 16946335 PMC1557968

[jonm13894-bib-0031] McPhail, S. M. (2016). Multimorbidity in chronic disease: Impact on health care resources and costs. Risk Management and Healthcare Policy, 9, 143–156. 10.2147/RMHP.S97248 27462182 PMC4939994

[jonm13894-bib-0032] Mosa, A. S. M. , Thongmotai, C. , Islam, H. , Paul, T. , Hossain, K. T. , & Mandhadi, V. (2021). Evaluation of machine learning applications using real‐world I data for predicting diabetes‐related long‐term complications. Journal of Business Analytics, 1–11. 10.1080/2573234X.2021.1979901

[jonm13894-bib-0033] Ng, Z. Q. P. , Ling, L. Y. J. , Chew, H. S. J. , & Lau, Y. (2021). The role of artificial intelligence in enhancing clinical nursing care: A scoping review. Journal of Nursing Management. 10.1111/jonm.13425 34272911

[jonm13894-bib-0034] Nickerson, H. D. , & Dutta, S. (2012). Diabetic complications: Current challenges and opportunities. Journal of Cardiovascular Translational Research, 5(4), 375–379. 10.1007/s12265-012-9388-1 22752737 PMC3396342

[jonm13894-bib-0035] Ozdemir, F. , Ari, A. , Kilcik, M. H. , Hanbay, D. , & Sahin, I. (2020). Prediction of neuropathy, neuropathic pain and kinesiophobia in patients with type 2 diabetes and design of computerized clinical decision support systems by using artificial intelligence. Medical Hypotheses, 143(12), 110070. 10.1016/j.mehy.2020.110070 32683220

[jonm13894-bib-0036] Ramesh, J. , Aburukba, R. , & Sagahyroon, A. (2021). A remote healthcare monitoring framework for diabetes prediction using machine learning. Healthcare Technology Letters, 8(3), 45–57. 10.1049/htl2.12010 34035925 PMC8136765

[jonm13894-bib-0037] Risling, T. (2017). Educating the nurses of 2025: Technology trends of the next decade. Nurse Education in Practice, 22, 89–92. 10.1016/j.nepr.2016.12.007 28049072

[jonm13894-bib-0038] Robert, N. (2019). How artificial intelligence is changing nursing. Nursing Management, 50(9), 30–39. 10.1097/01.NUMA.0000578988.56622.21 PMC7597764 31425440

[jonm13894-bib-0039] Seibert, K. , Domhoff, D. , Bruch, D. , Schulte‐Althoff, M. , Fürstenau, D. , Biessmann, F. , & Wolf‐Ostermann, K. (2021). Application scenarios for artificial intelligence in nursing care: Rapid review. Journal of Medical Internet Research, 23(11), e26522. 10.2196/26522 34847057 PMC8669587

[jonm13894-bib-0040] Shah, M. , & Vella, A. (2014). What is type 2 diabetes? Medicine, 42(12), 687–691. 10.1016/j.mpmed.2014.09.013

[jonm13894-bib-0041] Shang, Z. (2021). A concept analysis on the use of artificial intelligence in nursing. Cureus, 13(5), e14857. 10.7759/cureus.14857 34113496 PMC8177028

[jonm13894-bib-0042] Sharma, T. , & Shah, M. (2021). A comprehensive review of machine learning techniques on diabetes detection. Visual Computing for Industry, Biomedicine, and Art, 4(1), 1, 30–16. 10.1186/s42492-021-00097-7 34862560 PMC8642577

[jonm13894-bib-0043] Shi, R. , Niu, Z. , Wu, B. , Zhang, T. , Cai, D. , Sun, H. , Hu, Y. , Mo, R. , & Hu, F. (2020). Nomogram for the risk of diabetic nephropathy or diabetic retinopathy among patients with type 2 diabetes mellitus based on questionnaire and biochemical indicators: A cross‐sectional study. Diabetes, Metabolic Syndrome and Obesity: Targets and Therapy, 13, 1215–1229. 10.2147/DMSO.S244061 32368114 PMC7182465

[jonm13894-bib-0044] Singla, R. , Singla, A. , Gupta, Y. , & Kalra, S. (2019). Artificial intelligence/machine learning in diabetes care. Indian Journal of Endocrinology and Metabolism, 23(4), 495–497. 10.4103/ijem.IJEM_228_19 31741913 PMC6844177

[jonm13894-bib-0045] Svedberg, P. , Reed, J. , Nilsen, P. , Barlow, J. , Macrae, C. , & Nygren, J. (2022). Toward successful implementation of artificial intelligence in health care practice: Protocol for a research program. JMIR Research Protocols, 11(3), e34920. 10.2196/34920 35262500 PMC8943554

[jonm13894-bib-0046] Tahri Sqalli, M. , & Al‐Thani, D. (2020, September). On how chronic conditions affect the patient‐AI interaction: A literature review. Health, 8(3), 313. 10.3390/healthcare8030313 PMC7551169 32883036

[jonm13894-bib-0047] Tarumi, S. , Takeuchi, W. , Chalkidis, G. , Rodriguez‐Loya, S. , Kuwata, J. , Flynn, M. , Turner, K. M. , Sakaguchi, F. H. , Weir, C. , Kramer, H. , Shields, D. E. , Warner, P. B. , Kukhareva, P. , Ban, H. , & Kawamoto, K. (2021). Leveraging artificial intelligence to improve chronic disease care: Methods and application to pharmacotherapy decision support for Type‐2 diabetes mellitus. Methods of Information in Medicine, 60(1), e32–e43. 10.1055/s-0041-1728757 33975376 PMC8294941

[jonm13894-bib-0048] von Gerich, H. , Moen, H. , Block, L. J. , Chu, C. H. , DeForest, H. , Hobensack, M. , Michalowski, M. , Mitchell, J. , Nibber, R. , Olalia, M. A. , Pruinelli, L. , Ronquillo, C. E. , Topaz, M. , & Peltonen, L. M. (2021). Artificial intelligence‐based technologies in nursing: A scoping literature review of the evidence. International Journal of Nursing Studies, 127, 104153. 10.1016/j.ijnurstu.2021.104153 35092870

[jonm13894-bib-0049] Wang, H. , Xu, Y. , Chen, Q. , & Wang, X. (2021). Diagnosis of complications of type 2 diabetes based on weighted multi‐label small sphere and large margin machine. Applied Intelligence, 51(1), 223–236. 10.1007/s10489-020-01824-y

[jonm13894-bib-0050] Yousefi, L. , & Tucker, A. (2020). Predicting type 2 diabetes complications and personalising patient using artificial intelligence methodology. In: Type 2 Diabetes. IntechOpen.

[jonm13894-bib-0051] Zou, Q. , Qu, K. , Luo, Y. , Yin, D. , Ju, Y. , & Tang, H. (2018). Predicting diabetes mellitus with machine learning techniques. Frontiers in Genetics, 9, 515. 10.3389/fgene.2018.00515 30459809 PMC6232260

