# Supplementary material for: Artificial intelligence based prediction models for individuals at risk of multiple diabetic complications: A systematic review of the literature
Source: J Nurs Manag. 2022 Nov 23;30(8):3765–76. doi: 10.1111/jonm.13894 (PMC10100477; doi:10.1111/jonm.13894)
Supplement: Supplementary file 1 — Table S1. Search strategy (01.11.2021) Table S2. Outcomes of models for predicting diabetes‐related complications [file JONM-30-3765-s001.docx]

**Supplementary Material 1: Search strategy (01.11.2021)**

| **#** | **Keywords** | **PubMed** | **CINAHL** | **MEDLINE** | **Scopus** |
| --- | --- | --- | --- | --- | --- |
| 1 | "artificial intelligence" OR AI OR "machine learning" OR "deep learning" OR "data mining" OR "predictive models" OR "predictive modeling" OR "prediction model" OR "neural network" OR "deep learning" OR "decision tree" OR "random forest" OR "nearest neighbours" OR "support vector machines" OR "gbm" OR "gradient boosting" | 1.215.216 | 46.384 | 363.894 | 1.776.105 |
| 2 | "diabetes mellitus type 2" OR "type 2 diabetes" OR "type 2 diabetes mellitus" OR "non-insulin-dependent diabetes" OR "diabetes mellitus, noninsulin dependent" OR "diabetes mellitus, non-insulin-dependent" OR "diabetes mellitus, noninsulin-dependent" OR "adult-onset diabetes" OR "diabetes mellitus, adult-onset" OR "diabetes mellitus, ketosis-resistant" OR "diabetes mellitus, maturity-onset" OR "diabetes mellitus, slow-onset" OR "diabetes mellitus, stable" OR "maturity-onset diabetes" OR "maturity-onset diabetes mellitus" OR NIDDM OR T2DM OR "diabetic patient" | 211.533 | 88.026 | 208.624 | 345.908 |
| 3 | "prediction of complications" OR "prediction of diabetes complications" OR "diabetes mellitus complications" OR "retinopathy prediction" OR "diabetic foot prediction" OR "cardiovascular disease prediction" OR "nephropathy prediction" OR "neuropathy prediction" OR "hypoglycemia prediction" OR " hyperglycemia prediction" | 47.116 | 13.103 | 23.091 | 2.885 |
|  | #1 AND #2 AND #3 | 183 | 14 | 35 | 19 |

**Supplementary Material 2: Outcomes of models for predicting diabetes-related complications**

| Authors (year),  country | Diabetes-related complications | AUC (± SD) | CI | Accuracy (± SD) | Sensitivity (± SD) | Specificity (± SD) |
| --- | --- | --- | --- | --- | --- | --- |
| Aminian, et al., (2020), Ohio | All-cause mortality | Regression (metabolic surgery): 0.79  Regression (usual care): 0.81 | NA | Regression (metabolic surgery): 0.13  Regression (usual care): 0.24 | NA | NA |
|  | Coronary artery events | RF (metabolic surgery): 0.66  Regression (usual care): 0.67 | NA | RF (metabolic surgery): 0.03  Regression (usual care): 0.04 | NA | NA |
|  | Heart failure | Regression (metabolic surgery): 0.73  Regression (usual care): 0.75 | NA | Regression (metabolic surgery): 0.05  Regression (usual care): 0.14 | NA | NA |
|  | Nephropathy | Regression (metabolic surgery): 0.73  RF (usual care): 0.76 | NA | Regression (metabolic surgery): 0.07  RF (usual care): 0.14 | NA | NA |
| Dagliati, et al., (2018),  Italy | Nephropathy | SVM (3 years): 0.502  SVM (5 years): 0.502  SVM (7 years): 0.528 | SVM (3 years): 0.466-0.538  SVM (5 years): 0.461-0.542  SVM (7 years): 0.477-0.579 | LR:  0.647  0.693  0.686 | LR:  0.652  0.750  0.714 | LR:  0.642  0.616  0.643 |
|  |  | RF (3 years): 0.509  RF (5 years): 0.506  RF (7 years): 0.57 | RF (3 years): 0.488-0.529  RF (5 years): 0.484-0.528  RF (7 years): 0.525-0.615 |  |  |  |
|  |  | LR (3 years): 0.674  LR (5 years): 0.674  LR (7 years): 0.674 | LR (3 years): 0.594-0.754  LR (5 years): 0.615-0.756  LR (7 years): 0.594-0.736 |  |  |  |
|  |  | NB (3 years): 0.497  NB (5 years): 0.497  NB (7 years): 0.497 | NB (3 years): 0.495-0.5  NB (5 years): 0.494-0.56  NB (7 years): 0.518-0.618 |  |  |  |
|  | Neuropathy | SVM (3 years): 0.489  SVM (5 years): 0.503  SVM (7 years): 0.5 | SVM (3 years): 0.483-0.495  SVM (5 years): 0.472-0.533  SVM (7 years): 0.459-0.541 | LR:  0.746  0.680  0.727 | LR:  0.783  0.667  0.688 | LR:  0.707  0.697  0.780 |
|  |  | RF (3 years): 0.5  RF (5 years): 0.497  RF (7 years): 0.507 | RF (3 years): 0.5-0.5  RF (5 years): 0.493-0.501  RF (7 years): 0.474-0.54 |  |  |  |
|  |  | LR (3 years): 0.726  LR (5 years): 0.691  LR (7 years): 0.664 | LR (3 years): 0.614-0.837  LR (5 years): 0.59-0.792  LR (7 years): 0.568-0.761 |  |  |  |
|  |  | NB (3 years): 0.533  NB (5 years): 0.56  NB (7 years): 0.586 | NB (3 years): 0.473-0.592  NB (5 years): 0.498-0.623  NB (7 years): 0.522-0.651 |  |  |  |
|  | Retinopathy | SVM (3 years): 0.487  SVM (5 years): 0.513  SVM (7 years): 0.507 | SVM (3 years): 0.48-0.493  SVM (5 years): 0.472-0.554  SVM (7 years): 0.459-0.555 | LR:  0.777  0.743  0.666 | LR:  0.820  0.790  0.606 | LR:  0.730  0.685  0.745 |
|  |  | RF (3 years): 0.516  RF (5 years): 0.557  RF (7 years): 0.562 | RF (3 years): 0.483-0.548  RF (5 years): 0.507-0.607  RF (7 years): 0.51-0.614 |  |  |  |
|  |  | LR (3 years): 0.757  LR (5 years): 0.745  LR (7 years): 0.722 | LR (3 years): 0.66-0.854  LR (5 years): 0.656-0.834  LR (7 years): 0.629-0.814 |  |  |  |
|  |  | NB (3 years): 0.617  NB (5 years): 0.647  NB (7 years): 0.637 | NB (3 years): 0.536-0.697  NB (5 years): 0.536-0.697  NB (7 years): 0.566-0.707 |  |  |  |
| Fan, et al., (2021), China | Diabetic nephropathy | XF: 0.902 ± 0.040  CHAID: 0.699 ± 0.074  BN: 0.744 ± 0.078  D: 0.823 ± 0.055 | NA | XF: 0.862 ± 0.054  CHAID: 0.699 ± 0.067  BN: 0.916 ± 0.057  D: 0.720 ± 0.063 | NA | NA |
|  | Diabetic peripheral neuropathy | XF: 0.847 ± 0.081  CHAID: 0.787 ± 0.081  QUEST: 0.720 ± 0.060  D: 0.859 ± 0.050 | NA | XF: 0.783 ± 0.080  CHAID: 0.757 ± 0.054  QUEST: 0.766 ± 0.056  D: 0.843 ± 0.038 | NA | NA |
|  | Diabetic angiopathy | XF: 0.889 ± 0.059  CHAID: 0.764 ± 0.087  CRT: 0.797 ± 0.068  D: 0.825 ± 0.070 | NA | XF: 0.851 ± 0.051  CHAID: 0.769 ± 0.049  CRT: 0.802 ± 0.058  D: 0.808 ± 0.065 | NA | NA |
|  | Diabetic eye disease | ANN: 0.725 ± 0.142  CHAID: 0.818 ± 0.161  BN: 0.749 ± 0.179  D: 0.832 ± 0.086 | NA | ANN: 0.812 ± 0.091  CHAID: 0.875 ± 0.053  BN: 0.978 ± 0.031  D: 0.799 ± 0.055 | NA | NA |
| Kim, et al., (2019), Massachusetts | Ischemic Heart Disease | OLDW (internal – variant_OL.A1c_): 0.74  OLDW (internal – variant_OL.FPG_): 0.75  UMMC (internal – variant_UMMC_): 0.73  MCR (internal – variant_MCR_): 0.78 | OLDW (internal – variant_OL.A1c_): 0.73–0.87  OLDW (internal – variant_OL.FPG_): 0.73–0.87  UMMC (internal – variant_UMMC_): 0.71–0.94  MCR (internal – variant_MCR_): 0.70–0.94 | NA | NA | NA |
|  | Congestive Heart Failure | OLDW (internal – variant_OL.A1c_): 0.81  OLDW (internal – variant_OL.FPG_): 0.81  UMMC (internal – variant_UMMC_): 0.80  MCR (internal – variant_MCR_): 0.82 | OLDW (internal – variant_OL.A1c_): 0.73–0.87  OLDW (internal – variant_OL.FPG_): 0.73–0.87  UMMC (internal – variant_UMMC_): 0.71–0.94  MCR (internal – variant_MCR_): 0.68–0.95 | NA | NA | NA |
|  | Cerebrovascular Disease | OLDW (internal – variant_OL.A1c_): 0.74  OLDW (internal – variant_OL.FPG_): 0.74  UMMC (internal – variant_UMMC_): 0.75  MCR (internal – variant_MCR_): 0.73 | OLDW (internal – variant_OL.A1c_): 0.73–0.87  OLDW (internal – variant_OL.FPG_): 0.73–0.87  UMMC (internal – variant_UMMC_): 0.71–0.94  MCR (internal – variant_MCR_): 0.68–0.94 | NA | NA | NA |
|  | Peripheral Vascular Disease | OLDW (internal – variant_OL.A1c_): 0.75  OLDW (internal – variant_OL.FPG_): 0.74  UMMC (internal – variant_UMMC_): 0.73  MCR (internal – variant_MCR_): 0.75 | OLDW (internal – variant_OL.A1c_): 0.73–0.87  OLDW (internal – variant_OL.FPG_): 0.73–0.87  UMMC (internal – variant_UMMC_): 0.71–0.94  MCR (internal – variant_MCR_): 0.70–0.94 | NA | NA | NA |
|  | Chronic Kidney Disease | OLDW (internal – variant_OL.A1c_): 0.80  OLDW (internal – variant_OL.FPG_): 0.81  UMMC (internal – variant_UMMC_): 0.80  MCR (internal – variant_MCR_): 0.76 | OLDW (internal – variant_OL.A1c_): 0.73–0.87  OLDW (internal – variant_OL.FPG_): 0.73–0.87  UMMC (internal – variant_UMMC_): 0.71–0.94  MCR (internal – variant_MCR_): 0.70–0.94 | NA | NA | NA |
|  | Chronic Renal Failure | OLDW (internal – variant_OL.A1c_): 0.86  OLDW (internal – variant_OL.FPG_): 0.87  UMMC (internal – variant_UMMC_): 0.92  MCR (internal – variant_MCR_): 0.90 | OLDW (internal – variant_OL.A1c_): 0.73–0.87  OLDW (internal – variant_OL.FPG_): 0.73–0.87  UMMC (internal – variant_UMMC_): 0.71–0.94  MCR (internal – variant_MCR_): 0.70–0.95 | NA | NA | NA |
| Lagani, et al., (2015), Greece | CVD-DCCT | T1DM (internal): 0.7257  T1DM (external): 0.6887  T2DM (external): 0.7143 | T1DM (internal): 0.50962–0.8629  T1DM (external): 0.4923–0.86207  T2DM (external): 0.62384–0.80563 | NA | NA | NA |
|  | CVD-EDIC | T1DM (internal): 0.6204  T1DM (external): 0.4862  T2DM (external): 0.6099 | T1DM (internal): 0.5549–0.69224  T1DM (external): 0.18084–0.81984  T2DM (external): 0.50211–0.71809 | NA | NA | NA |
|  | Hypoglycemia | T1DM (internal): 0.6694  T1DM (external): 0.6903  T2DM (external): 0.7002 | T1DM (internal): 0.58766–0.75118  T1DM (external): 0.5–0.8691  T2DM (external): 0.19012–0.97115 | NA | NA | NA |
|  | Ketoacidosis | T1DM (internal): 0.6745  T1DM (external): 0.8182  T2DM (external): - | T1DM (internal): 0.59412–0.75479  T1DM (external): 0.23077–1  T2DM (external): - | NA | NA | NA |
|  | Microalbuminuria | T1DM (internal): 0.7421  T1DM (external): 0.824  T2DM (external): 0.5701 | T1DM (internal): 0.6751–0.77652  T1DM (external): 0.66234–0.96875  T2DM (external): 0.52144–0.62193 | NA | NA | NA |
|  | Preteinuria | T1DM (internal): 0.8330  T1DM (external): -  T2DM (external): 0.6569 | T1DM (internal): 0.53521–0.96223  T1DM (external): -  T2DM (external): 0.53261–0.77125 | NA | NA | NA |
|  | Neuropathy | T1DM (internal): 0.6661  T1DM (external): 0.735  T2DM (external): 0.4359 | T1DM (internal): 0.54626–0.74187  T1DM (external): 0.55102–0.90754  T2DM (external): 0.32132–0.56216 | NA | NA | NA |
| Lee, et al., (2021), China | Mortality | RSF: 0.8377  Cox: 0.7221 | NA | NA | NA | NA |
|  | Renal | RSF: 0.8577  Cox: 0.8008 | NA | NA | NA | NA |
|  | Peripheral vascular disease | RSF: 0.8517  Cox: 0.7517 | NA | NA | NA | NA |
|  | Neurological | RSF: 0.8480  Cox: 0.7969 | NA | NA | NA | NA |
|  | Ophthalmological | RSF: 0.8643  Cox: 0.7814 | NA | NA | NA | NA |
|  | Ischemic stroke | RSF: 0.8634  Cox: 0.7884 | NA | NA | NA | NA |
|  | Atrial fibrillation | RSF: 0.8523  Cox: 0.7742 | NA | NA | NA | NA |
|  | Heart failure | RSF: 0.8330  Cox: 0.7585 | NA | NA | NA | NA |
|  | Intracranial hemorrhage | RSF: 0.7034  Cox: 0.6857 | NA | NA | NA | NA |
|  | Ischemic heart disease | RSF: 0.8328  Cox: 0.7985 | NA | NA | NA | NA |
|  | Acute myocardial infarction | RSF: 0.8246  Cox: 0.7499 | NA | NA | NA | NA |
|  | Osteoporosis | RSF: 0.7372  Cox: 0.6890 | NA | NA | NA | NA |
|  | Dementia | RSF: 0.8549  Cox: 0.7345 | NA | NA | NA | NA |
|  | Retinopathy | T1DM (internal): 0.6564  T1DM (external): 0.7201  T2DM (external): 0.5451 | T1DM (internal): 0.60826–0.6745  T1DM (external): 0.58669–0.8745  T2DM (external): 0.47399–0.6189 | NA | NA | NA |
| Liu, et al., (2020), China | Diabetic nephropathy (DN) | BN: 0.831  BN-wopi: 0.83  NB: 0.826  RF: 0.83  C5.0: 0.806 | BN: 0.7947–0.8665  BN-wopi: 0.7931–0.866  NB: 0.7765–0.8761  RF: 0.7747–0.8857  C5.0: 0.7481–0.8639 | NA | BN: 0.86  BN-wopi: 0.511  NB: 0.727  RF: 0.694  C5.0: 0.756 | BN: 0.655  BN-wopi: 0.959  NB: 0.788  RF: 0.906  C5.0: 0.826 |
|  | Diabetic foot (DF) | BN: 0.905  BN-wopi: 0.788  NB: 0.704  RF: 0.761  C5.0: 0.851 | BN: 0.8841–0.9268  BN-wopi: 0.5109–1  NB: 0.5531–0.8545  RF: 0.555–0.9664  C5.0: 0.7532–0.9496 | NA | BN: 1  BN-wopi: 0.833  NB: 1  RF: 0.833  C5.0: 1 | BN: 0.891  BN-wopi: 0.884  NB: 0.541  RF: 0.604  C5.0: 0.602 |
|  | Diabetic macrovascular complications (DMV) | BN: 0.753  BN-wopi: 0.749  NB: 0.723  RF: 0.745  C5.0: 0.726 | BN: 0.6861–0.8193  BN-wopi: 0.7056–0.7916  NB: 0.6615–0.7841  RF: 0.6693–0.8198  C5.0: 0.6992–0.7524 | NA | BN: 0.827  BN-wopi: 0.855  NB: 0.861  RF: 0.765  C5.0: 0.804 | BN: 0.563  BN-wopi: 0.584  NB: 0.503  RF: 0.633  C5.0: 0.599 |
|  | Diabetic peripheral neuropathy (DPN) | BN: 0.545  BN-wopi: 0.685  NB: 0.505  RF: 0.516  C5.0: 0.527 | BN: 0.4129–0.6767  BN-wopi: 0.5885–0.7807  NB: 0.04515–0.9649  RF: 0.3111–0.7219  C5.0: 0.4238–0.6305 | NA | BN: 0.75  BN-wopi: 0.875  NB: 0.5  RF: 0.2  C5.0: 0.5 | BN: 0.475  BN-wopi: 0.457  NB: 0.827  RF: 1  C5.0: 0.554 |
|  | Diabetic ketoacidosis (DK) | BN: 0.877  BN-wopi: 0.898  NB: 0.876  RF: 0.858  C5.0: 0.819 | BN: 0.8182–0.9362  BN-wopi: 0.8553–0.9402  NB: 0.7875–0.9651  RF: 0.7665–0.949  C5.0: 0.7354–0.9018 | NA | BN: 0.867  BN-wopi: 0.875  NB: 0.688  RF: 0.619  C5.0: 0.606 | BN: 0.76  BN-wopi: 0.817  NB: 0.917  RF: 0.968  C5.0: 0.912 |
| Ljubic, et al., (2020), Pennsylvania | Angina pectoris | NA | NA | GRU RNN (4-visit): 0.796 ± 0.024  Bidirectional GRU (4-visit): 0.796 ± 0.024  Bidirectional GRU (3-visit): 0.789 ± 0.012  Bidirectional GRU (2-visit): 0.738 ± 0.016  1-way LSTM (4-visit): 0.780 ± 0.016  1-way LSTM (3-visit): 0.793 ± 0.019  1-way LSTM (2-visit): 0.738 ± 0.018  RF (4-visit): 0.717 ± 0.011  RF (3-visit): 0.722 ± 0.012  RF (2-visit): 0.701 ± 0.013  MLP (4-visit): 0.743 ± 0.013  MLP (3-visit): 0.732 ± 0.008  MLP (2-visit): 0.714 ± 0.009 | GRU RNN (4-visit): 0.796 ± 0.024 | GRU RNN (4-visit):  0.796 ± 0.024 |
|  | Atherosclerosis | NA | NA | GRU RNN (4-visit): 0.756 ± 0.003  Bidirectional GRU (4-visit): 0.756 ± 0.003  Bidirectional GRU (3-visit): 0.750 ± 0.008  Bidirectional GRU (2-visit): 0.713 ± 0.011  1-way LSTM (4-visit): 0.750 ± 0.015  1-way LSTM (3-visit): 0.745 ± 0.012  1-way LSTM (2-visit): 0.701 ± 0.018  RF (4-visit): 0.712 ± 0.007  RF (3-visit): 0.704 ± 0.011  RF (2-visit): 0.689 ± 0.014  MLP (4-visit): 0.691 ± 0.008  MLP (3-visit): 0.671 ± 0.008  MLP (2-visit): 0.665 ± 0.012 | GRU RNN (4-visit): 0.791 ± 0.012 | GRU RNN (4-visit): 0.718 ± 0.014 |
|  | Ischemic chronic heart disease (ICHD) | NA | NA | GRU RNN (4-visit): 0.835 ± 0.005  Bidirectional GRU (4-visit): 0.835 ± 0.005  Bidirectional GRU (3-visit): 0.814 ± 0.008  Bidirectional GRU (2-visit): 0.802 ± 0.010  1-way LSTM (4-visit): 0.828 ± 0.008  1-way LSTM (3-visit): 0.813 ± 0.007  1-way LSTM (2-visit): 0.802 ± 0.015  RF (4-visit): 0.759 ± 0.009  RF (3-visit): 0.745 ± 0.010  RF (2-visit): 0.744 ± 0.014  MLP (4-visit): 0.761 ± 0.017  MLP (3-visit): 0.763 ± 0.015  MLP (2-visit): 0.758 ± 0.015 | GRU RNN (4-visit): 0.886 ± 0.014 | GRU RNN (4-visit): 0.787 ± 0.012 |
|  | Depressive disorder | NA | NA | GRU RNN (4-visit): 0.820 ± 0.005  Bidirectional GRU (4-visit): 0.820 ± 0.005  Bidirectional GRU (3-visit): 0.802 ± 0.018  Bidirectional GRU (2-visit): 0.773 ± 0.021  1-way LSTM (4-visit): 0.812 ± 0.008  1-way LSTM (3-visit): 0.810 ± 0.004  1-way LSTM (2-visit): 0.776 ± 0.019  RF (4-visit): 0.714 ± 0.011  RF (3-visit): 0.739 ± 0.014  RF (2-visit): 0.722 ± 0.016  MLP (4-visit): 0.752 ± 0.013  MLP (3-visit): 0.741 ± 0.015  MLP (2-visit): 0.761 ± 0.016 | GRU RNN (4-visit): 0.848 ± 0.009 | GRU RNN (4-visit): 0.792 ± 0.010 |
|  | Hearing impairment | NA | NA | GRU RNN (4-visit): 0.734 ± 0.017  Bidirectional GRU (4-visit): 0.734 ± 0.017  Bidirectional GRU (3-visit): 0.743 ± 0.017  Bidirectional GRU (2-visit): 0.716 ± 0.019  1-way LSTM (4-visit): 0.720 ± 0.021  1-way LSTM (3-visit): 0.730 ± 0.020  1-way LSTM (2-visit): 0.694 ± 0.022  RF (4-visit): 0.691± 0.019  RF (3-visit): 0.694 ± 0.021  RF (2-visit): 0.680 ± 0.024  MLP (4-visit): 0.701 ± 0.021  MLP (3-visit): 0.704 ± 0.019  MLP (2-visit): 0.671 ± 0.023 | GRU RNN (4-visit): 0.743 ± 0.016 | GRU RNN (4-visit): 0.792 ± 0.010 |
|  | Myocardial infarction (MI) | NA | NA | GRU RNN (4-visit): 0.733 ± 0.011  Bidirectional GRU (4-visit): 0.733 ± 0.011  Bidirectional GRU (3-visit): 0.723 ± 0.014  Bidirectional GRU (2-visit): 0.711 ± 0.015  1-way LSTM (4-visit): 0.713 ± 0.013  1-way LSTM (3-visit): 0.701 ± 0.012  1-way LSTM (2-visit): 0.679 ± 0.013  RF (4-visit): 0.691 ± 0.010  RF (3-visit): 0.688 ± 0.013  RF (2-visit): 0.663 ± 0.015  MLP (4-visit): 0.661 ± 0.016  MLP (3-visit): 0.665 ± 0.014  MLP (2-visit): 0.662 ± 0.017 | GRU RNN (4-visit): 0.806 ± 0.021 | GRU RNN (4-visit): 0.652 ± 0.012 |
|  | Nephropathy | NA | NA | GRU RNN (4-visit): 0.768 ± 0.012  Bidirectional GRU (4-visit): 0.768 ± 0.012  Bidirectional GRU (3-visit): 0.768 ± 0.012  Bidirectional GRU (2-visit): 0.742 ± 0.008  1-way LSTM (4-visit): 0.742 ± 0.008  1-way LSTM (3-visit): 0.748 ± 0.013  1-way LSTM (2-visit): 0.738 ± 0.010  RF (4-visit): 0.699 ± 0.014  RF (3-visit): 0.696 ± 0.015  RF (2-visit): 0.695 ± 0.012  MLP (4-visit): 0.694 ± 0.024  MLP (3-visit): 0.689 ± 0.020  MLP (2-visit): 0.678 ± 0.015 | GRU RNN (4-visit): 0.826 ± 0.017 | GRU RNN (4-visit): 0.654 ± 0.021 |
|  | Neuropathy | NA | NA | GRU RNN (4-visit): 0.746 ± 0.053  Bidirectional GRU (4-visit): 0.746 ± 0.053  Bidirectional GRU (3-visit): 0.738 ± 0.043  Bidirectional GRU (2-visit): 0.715 ± 0.038  1-way LSTM (4-visit): 0.719 ± 0.073  1-way LSTM (3-visit): 0.739 ± 0.068  1-way LSTM (2-visit): 0.712 ± 0.054  RF (4-visit): 0.671 ± 0.033  RF (3-visit): 0.664 ± 0.040  RF (2-visit): 0.660 ± 0.035  MLP (4-visit): 0.668 ± 0.039  MLP (3-visit): 0.664 ± 0.046  MLP (2-visit): 0.662 ± 0.055 | GRU RNN (4-visit): 0.795 ± 0.041 | GRU RNN (4-visit): 0.701 ± 0.049 |
|  | Peripheral vascular disease (PVD) | NA | NA | GRU RNN (4-visit): 0.767 ± 0.002  Bidirectional GRU (4-visit): 0.767 ± 0.002  Bidirectional GRU (3-visit): 0.759 ± 0.006  Bidirectional GRU (2-visit): 0.738 ± 0.011  1-way LSTM (4-visit): 0.744 ± 0.014  1-way LSTM (3-visit): 0.743 ± 0.010  1-way LSTM (2-visit): 0.738 ± 0.014  RF (4-visit): 0.695 ± 0.006  RF (3-visit): 0.708 ± 0.009  RF (2-visit): 0.701 ± 0.008  MLP (4-visit): 0.691 ± 0.014  MLP (3-visit): 0.684 ± 0.010  MLP (2-visit): 0.680 ± 0.012 | GRU RNN (4-visit): 0.774 ± 0.005 | GRU RNN (4-visit): 0.753 ± 0.011 |
|  | Retinopathy | NA | NA | GRU RNN (4-visit): 0.796 ± 0.014  Bidirectional GRU (4-visit): 0.796 ± 0.014  Bidirectional GRU (3-visit): 0.752 ± 0.021  Bidirectional GRU (2-visit): 0.728 ± 0.019  1-way LSTM (4-visit): 0.782 ± 0.001  1-way LSTM (3-visit): 0.731 ± 0.013  1-way LSTM (2-visit): 0.725 ± 0.014  RF (4-visit): 0.741 ± 0.011  RF (3-visit): 0.698 ± 0.012  RF (2-visit): 0.696 ± 0.018  MLP (4-visit): 0.740 ± 0.007  MLP (3-visit): 0.700 ± 0.011  MLP (2-visit): 0.676 ± 0.012 | GRU RNN (4-visit): 0.799 ± 0.007 | GRU RNN (4-visit): 0.792 ± 0.018 |
| Ozdemir, et al., (2020), Turkey | Neuropathy | 0.99 | NA | 0.99 | 0.99 | 0.99 |
|  | Neuropathic pain | 0.99 | NA | 0.99 | 0.99 | 0.99 |
|  | Kinesiophobia | 0.99 | NA | 0.99 | 0.99 | 0.99 |
| Shi, et al., (2020), China | Diabetic nephropathy (DN), diabetic retinopathy (DR) | *  0.807 | *  0.784–0.830 | NA | NA | NA |
| Wang, et al., (2021), China | Macrovascular, microvascular, and neuropathy | NA | NA | *****  BR (linear): 0.685 ± 0.000  BR (poly): 0.701 ± 0.000  BR (rbf): 0.701 ± 0.000  WML-SSLM (linear): 0.520 ± 0.001  WML-SSLM (poly): 0.661 ± 0.002  WML-SSLM (rbf): 0.697 ± 0.002 | NA | NA |

* Multi-label classifier metric was used to estimate the accuracy of covering all three complications at once

*Legend: AUC=area under the curve, BN=Bayesian network model, BR=binary relevance, CI=confidence interval, CHAID= chi-squared automatic interaction detector, CVD=Cardiovascular Diseases, D=discriminate model, DCCT=Diabetes Control and Complications Trial, EDIC=Epidemiology of Diabetes Interventions and Complications, GRU=gated recurrent unit, LR= logistic regression, LSTM=long short-term memory, MCR=Mayo Clinic, Rochester, MLP= multilayer perceptron, NA=not available, NB= Naïve Bayes model, OLDW=OptumLabs Data Warehouse, RF=random forest, RNN= recurrent neural network, RSF=Random survival forests, SD=standard deviation, SVM=support vector machines, T1DM=type 1 diabetes mellitus, T2DM=type 2 diabetes mellitus, UMMC=University of Minnesota Medical Center, WML-SSL=weighted multi-label small sphere and large margin machine, QUEST=quick unbiased efficient statistical tree, XF=ensemble model*
